# Titanium Nickelide in Midface Fractures Treatment

**DOI:** 10.3390/jfb11030052

**Published:** 2020-07-27

**Authors:** Liudmila Shamanaeva, Ekaterina Diachkova, Pavel Petruk, Kirill Polyakov, Igor Cherkesov, Sergei Ivanov

**Affiliations:** 1 Department of Maxillofacial Surgery, I.M. Sechenov First Moscow State Medical University (Sechenov University), 119991 Moscow, Russia; petruk_pavel@yahoo.com (P.P.); 79067170999@yandex.ru (K.P.); cherkesovi@gmail.com (I.C.); syivanov@yandex.ru(S.I.); 2 Department of Oral Surgery, I.M. Sechenov First Moscow State Medical University (Sechenov University), 119991 Moscow, Russia; secu2003@mail.ru; 3 Department of Maxillofacial and Oral Surgery, Peoples Friendship University of Russia (RUDN University), 117198 Moscow, Russia

**Keywords:** titanium nickelide, orbital floor, zygoma fracture, midface fracture, endoprosthesis

## Abstract

Background: The question of reconstruction of human tissues and organs with the use of medical materials is still open, because of the accurate requirements for their biological and physical features. The aim of this study was to prove the efficiency of titanium nickelide constructors in treatment of isolated orbital floor fractures or combination with zygomatico-orbital complex fractures. Methods: Patients with a fracture of zygomatico-orbital complex and/or low orbital floor (n = 44) carried out different treatments: in the first group, osteosynthesis and endoprosthesis with titanium nickelide structures; in the second group, titan mini-plates osteosynthesis; in the third group (‘blow-out’), endoprosthesis with a titanium nickelide mesh; and in the fourth group (‘blow-out’), conservative treatment and monitoring (archive data) (*p* > 0.05). The paraesthesia, diplopia, enophthalmos and exophthalmos degree were measured in points. Results: In one year, the first and second groups had no differences in level of paraesthesia (*p* > 0.05). The absence of exophthalmos and differences between first and second groups, and between the third and the fourth groups with positive dynamics inside the groups were proved (*p* < 0.05). In the first and third groups, enophthalmos was absent, and it increased in the second and fourth groups (*p* < 0.01, *p* < 0.11). Diplopia in the first and third groups was absent, and it increased in the second and fourth groups (*p* < 0.05, *p* < 0.01). Conclusion: The elasticity and biocompatibility of titanium nickelide make the implant insertion and restoration of the lower orbital wall anatomy easier, with good postoperative clinical results.

## 1. Introduction

Today, metals and alloys are used as the most important functional elements in various fields of medicine [1,2,3]. Scientists are improving the properties of metals and alloys, and they also want to bring the characteristics of implants closer to the properties of body tissues [4,5,6,7].

Among the injuries of the facial skull zygomatic complex fractures occur in 25–30% of cases [8,9]. Statistical analysis shows that traffic collisions (41%), domestic accidents (23%) and sport accidents (18%) are main causes. Most of the patients suffering from craniofacial and skull base fractures are between 20 and 40 years of age. Men are significantly more involved in craniofacial trauma than women. Midface fractures frequently lead to fractures of the orbital walls. In cases of fragments dislocation, osteosynthesis is indicated [10]. Surgical treatment should be preferred, due to the patient’s early rehabilitation and the prevention of complications [11,12,13].

Unique medical technologies were created today based on the discovery of the hysteretic behaviour of biological tissues, and a new generation of biocompatible materials and shape memory implants has been developed [14]. Titanium nickelide implants are successfully used in clinical practice for orbital walls reconstruction and osteosynthesis, because this material is highly resistant to corrosion, has good biocompatibility with tissues (or is well biocompatible with tissues, relatively inexpensive, easy to model based on individual anatomy [15,16].

An alternative for titanium plates is the use of fixing structures made of alloys with the shape memory effect. In this regard, the issues in the development of new modalities for reconstructive treatment in zygomatic complex fractures remain extremely actual. Analysis of the properties of metallic materials and alloys shows that alloys based on nickel-titanium alloys are the most compatible materials with body tissues, which is due to the delay of biological systems [17].

**Figure 1:**
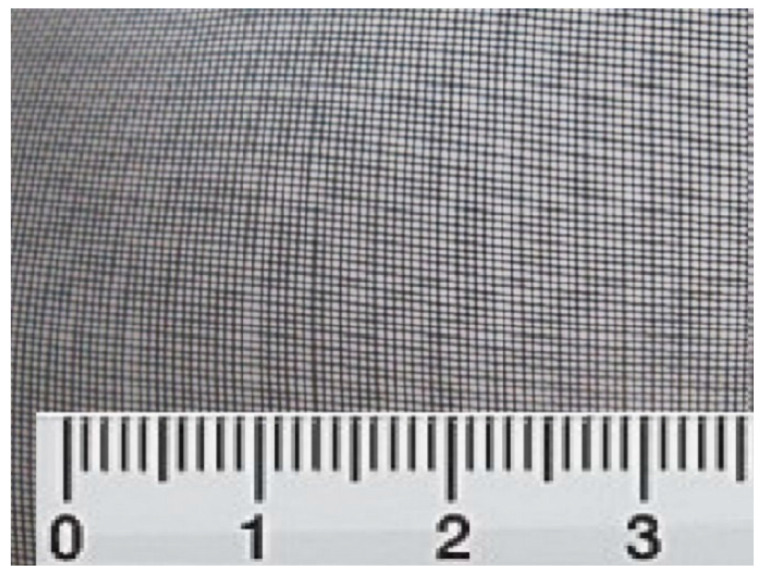
Shape memory titanium nickelide implant.

We used, in our research material for endoprosthesis of the orbital floor, a system of titanium nickelide filaments with an exact diameter of 60–80 microns. The distance between the threads is 180 microns (Figure 1). Implants in the form of rectangles 3.0 × 4.0 cm in size were prepared from the mesh tissue and sterilised.

The positive properties of this material are its elasticity, which makes it easy to insert an implant into the orbit and restore the anatomy of its lower wall. High bio-inertness, the ability to not cause immune reactions and inflammatory processes and the mesh structure of the implant contribute to the ‘germination’ of tissues and its good fixation, preventing displacement [18].

The aim of this study was to prove the efficiency of titanium nickelide constructors in the treatment of isolated orbital floor fractures or a combination with zygomatico-orbital complex fractures.

## 2. Materials and Methods 

We treated patients with a fracture of the zygomatico-orbital complex with displacement of fragments and a fracture of the lower orbital wall in the Department of Maxillofacial Surgery of Sechenov University for eight years from the year 2011.

The stages of the study were approved by the Ethical Committee of the Sechenov First Moscow State Medical University (protocol No. 02-12 dated by 12 November 2012). The written consent of all patients to use the results of research and computed tomograms was obtained.

Inclusion criteria:

1. Age of patients 18 years and older.
2. Traumatic zygomatico-orbital fracture with a fracture of the lower orbital wall.
3. Fracture of the lower orbital wall with displacement of fragments, changes in the parameters of the orbit and maxillary sinus.
4. Dysfunction of the eye (a change in the mobility and position of the eyeball, diplopia).
5. The period from the moment of injury to admission to the hospital is not more than 10 days.
6. The absence of severe somatic diseases.

Criteria for not including patients in our research:

1. Age up to 18 years.
2. Traumatic zygomatico-orbital fracture without a fracture of the lower orbital wall with displacement of fragments.
3. The period from the moment of injury to admission to the hospital is more than 10 days.
4. The presence of severe somatic diseases.

Exclusion criterion: Patient violation of recommendations in the postoperative period.

We divided patients into four groups:

First group—patients with a zygomatico-orbital fracture and a fracture of the lower orbital wall with displacement of fragments. We performed osteosynthesis and prosthetics of the lower orbital wall with a mesh of titanium nickelide.

Second group—archived data of patients with the same diagnosis, osteosynthesis without prosthetics of the orbit walls was made.

Third group—patients with an isolated fracture of the lower wall of the orbit (blow-out). We performed endoprosthesis with a titanium nickelide mesh.

Fourth group—archived data of patients with an isolated fracture of the lower wall of the orbit (blow-out), conservative treatment and dynamic observation without surgery were performed.

The patients’ age in all 4 groups varied from 18 to 47 years. The mean age in the 1st group was 30.2 ± 9.3 years; in the 2nd group it was 33.3 ± 8.9 years; in the 3rd group it was 31.3 ± 8.4 years; and in the 4th group it was 30.8 ± 9.9 years. The differences in age in all groups of patients were not significant (H = 0.93, *p* > 0.05).

Based on medical history the cause of trauma was mainly domestic or sport injury. We coded the cause in all four groups alike: 0 point for sport trauma and 1 point for domestic injury. The mean point in the 1st group was 0.8 ± 0.3; in the 2nd group it was 0.9 ± 0.4; in the 3rd group it was 0.55 ± 0.5; and in the 4th group, it was 0.64 ± 0.5. The differences in cause of trauma in all groups of patients were not significant (f-ratio = 1.63, *p* > 0.05).

All patients applied in hospital and the Department of Maxillofacial Surgery not later than 10 days after trauma. This period varied from one to five days in all groups. The average day of patient admission in the 1st group was 2.1 ± 1.2; in the 2nd group it was 2.2 ± 1.1; in the 3rd group it was 1.8 ± 1; and in the 4th group, it was 1.9 ± 1. The differences in days of patient admission in all groups were not significant (H = 1, *p* > 0.05).

The main characteristics of all four groups are described in Table 1.

We applied the patient examination algorithm:

1. Complaints, anamnesis of life and disease;
2. External examination and palpation;
3. X-ray examination and skull multispiral computed tomography with 3D reconstruction;
4. Consultation of neurologist and ophthalmologist;
5. Performing reconstructive surgery;
6. Monitoring and antibacterial and anti-inflammatory therapy;
7. Hospital discharge with recommendations.

During clinical examination, we consider some symptoms in points for better evaluation:

Paraesthesia: from 0 to 2, where 0 is the absence of paraesthesia, 1 is not significant paraesthesia and 2 is rather severe paraesthesia (the absence of sensitivity of examined area).

Diplopia: from 0 to 1, where 0 is the absence of diplopia and 1 is diplopia during gazing upward. The causes of the injury were coded by us: household—1, sports—0.

We performed multispiral computed tomography of the middle zone of the face, with 3D reconstruction for the diagnosis and determination of surgery stages.

We evaluated the position of the bone fragments, eyeball and periorbital fibre, eye muscles and optic nerve, the presence and size of the defect of the lower wall of the orbit, the signs of haemorrhage and the condition of the mucous membrane of the maxillary sinuses and ethmoid labyrinth. We paid special attention to the oculomotor muscles, and the prolapse of the contents of the orbit into the maxillary sinus. Analysis of computed tomograms was carried out in three projections: frontal, axial and sagittal. It should be noted that, in some cases, the X-ray picture was different from the clinical situation detected during surgery.

When studying control images in the postoperative period, we paid attention to the quality of repair and fixation of bone fragments, the position of the titanium nickelide implant and the quality of restoration of the lower orbital wall. Control computed tomography of the middle zone of the face was performed while the patient was in the hospital and one year after surgery.

All patients were consulted by an ophthalmologist and a neurologist, and drug therapy was carried out. Ophthalmological examination consisted in determining the acuity and fields of vision, haemorrhage and diplopia and examination of the fundus. Computed tomography was used to measure exophthalmos and enophthalmos [19,20].

### 2.1. Characteristics of Titanium Nickelide Implants

The material for endoprosthesis of orbital floor is a system of titanium nickelide filaments with a diameter of 60–80 microns. The distance between the threads is 180 microns. Implants in the form of rectangles 3.0 × 4.0 cm in size were prepared from the mesh tissue and sterilised.

The positive properties of this material are its elasticity, which makes it easy to insert an implant into the orbit and restore the anatomy of its lower wall, high bio-inertness, the ability to not cause immune reactions and inflammatory processes and the mesh structure of the implant contribute to the ‘germination’ of tissues and its good fixation, preventing displacement [11]. We have obtained the patent ‘Method for restoring the lower wall of the orbit and lower eye region after injury’ RU-2-486-872-C1-10.07.2013.

All patients gave their oral and written consent for surgery, examination, investigations and use of results for research. Before the operation, a stereolithographic model of the middle region of the face was made for all patients of the 1st and 3rd groups.

### 2.2. Operation Technique with Titanium Nickelide Mesh

**Figure 2:**
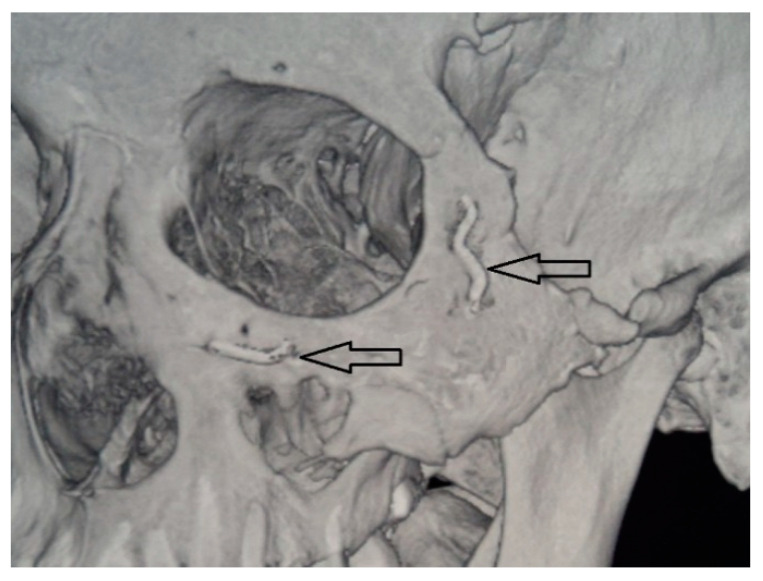
Titanium nickelide fixators.

After performing operative approaches and repositioning the zygomatic bone to the correct anatomical position, we fixed the zygomatic bone in the zygomatico-frontal suture, using titanium nickelide fixators with shape memory with compression 7H (Newton) and 14H or titanium microplate (Figure 2). Then, fragments were fixed in the region of the lower edge of the orbit. Then, from the side of the oral cavity the maxillary sinus was revised, and fragments were fixed along the lines of the conformers. A Foley catheter was placed in the maxillary sinus, which was filled under visual control of the lower orbital wall. Thereafter, mucous membrane Vicryl 4.0 was sutured.

**Figure 3:**
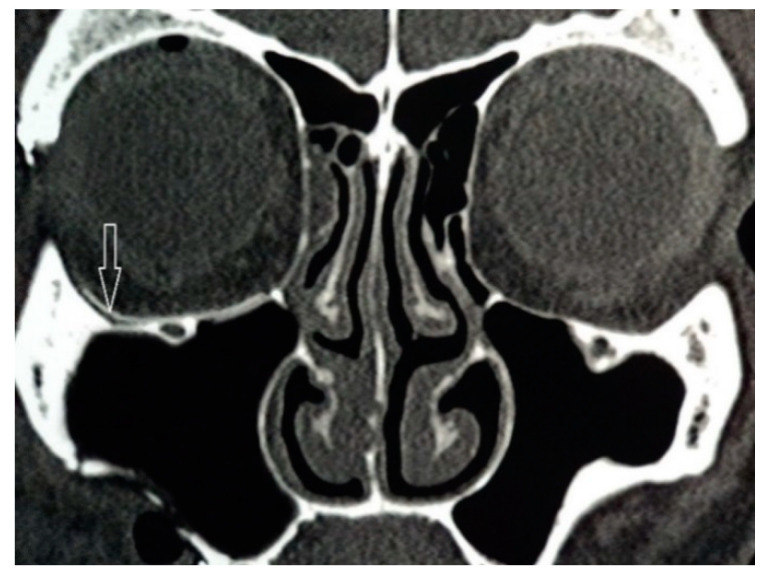
Shape memory titanium nickelide implant in orbital floor.

After fixing the zygomatic bone at three points, the lower orbital wall was again examined for bone defects, because the defect after repositioning the zygomatic bone could increase in size. After that, the implant was modelled intraoperatively using surgical scissors from the tissue of super-elastic titanium nickelide. Then, it was introduced into the orbit and installed in the correct anatomical position (Figure 3). The size of the endoprosthesis must exceed the size of the defect by at least 5 mm. Additional implant fixation was not required. The implant took the appropriate shape and held firmly in the tissues. All this made it possible to reduce the time of surgery and anaesthesia.

### 2.3. Statistics

The calculated R sample size was equal to 10. According to the possible dropout, the size of each group in our research was enlarged to 11 patients.

Before surgical treatment we assumed

**Theorem** **1.** *The diagnosis of fractures of the lower orbital wall and timely reconstruction prevents long-term complications associated with changes in the position of the eyeball, such as enophthalmos and diplopia.*

The null hypothesis µ0 was “there are no differences between group 1, where the nickelide titanium clamps were used for osteosynthesis and nickelide titanium endoprosthesis, and group 2 with the traditional surgical method of treatment”, and “there are no differences between group 3, where the nickelide titanium endoprosthesis was used, and group 2 with the conservative treatment and monitoring”.

The alternative hypothesis µ1 was “there are differences between group 1 and group 2 and groups 3 and 4 in the results of different algorithms of treatment in patients with trauma of zygomatico-orbital complex and fracture of low orbital wall required in reconstruction”.

We supposed µ0 is not equal to µ1.

We counted means, variances, standard error and statistical significance for all groups and their possible connections. We checked groups for normality of distribution; when the differences in means and variances did not allow us to use parametric statistics (one-way ANOVA), we used tests for nonparametric statistics (Kruskal-Wallis test). For comparing the differences between the main and control groups in pairs, we used the Mann-Whitney test; for the assessment of the dynamics of any criterion, we used x2 Pearson tables; and for the testing of the connection between different symptoms and the density of this correlation, we counted Pearson and Spearman coefficients (r and rs). For statistics, we used program R (RStudio Inc., Delaware Public Benefit Corporation, Wilmington, DE, USA, GNU Affero General Public License v3, ver.3.6.0 (2019-04-26)).

## 3. Results

In the first group, in 1 year after surgical treatment there was a statistically significant decreasing of the level of paraesthesia (U = 10, *p* < 0.01), exophthalmos (U = 11, *p* < 0.01) and diplopia (U = 27.5, *p* < 0.05) in comparison to pre-operative condition. Additionally, it was shown that there was a decrease in the level of enophthalmos, but without statistical significance (U = 60.5 and U = 49.5, *p* > 0.05).

In the second group, after 1 year, there was a decrease in the level of paraesthesia (U = 20.5, *p* < 0.01) and exophthalmos (U = 16.5, *p* < 0.01), in comparison to pre-operative condition. Additionally, it was shown the increase in the level of enophthalmos and the absence of positive dynamics of diplopia was without statistical significance (U = 38.5 and U = 60.5, *p* > 0.05).

In the third group, 1 year after operation, the level of exophthalmos (U = 5.5, *p* < 0.01) and diplopia (U = 27.5, *p* < 0.05) decreased, as did enophthalmos, but that was without statistical significance (U = 55, *p* > 0.05).

In the fourth group, after 1 year, a decrease in the level of exophthalmos (U = 11, *p* < 0.01) was registered, and a progression of enophthalmos (U = 19, *p* < 0.01), as well as an increase in the level of diplopia (U = 49.5, *p* > 0.05), but without statistical significance. 

Before operation, the appearance of paraesthesia in first and second group was approximately equal (1.09; 0–2 and 1; 0–2) (U = 56.5, *p* > 0.05). In 30 days after operation, the means of paraesthesia test were not changed, in the first group 0.55(0–1) and 0.55 (0–2) (U = 58, *p* > 0.05). One-year control examination has shown the absence of differences in level of paraesthesia between groups 0.09 (0–1) and 0.09 (0–1) (U = 60.5, *p* > 0.05) but positive dynamics inside them (*p* > 0.05).

There were no signs of paraesthesia in the third and fourth groups of research.

Before operation, the appearance of exophthalmos in first and second groups were approximately equal (1.36; 0–3 and 1.09; 0–3) (U = 51.5, *p* > 0.05). In 30 days after operation, the means of exophthalmos test were equal, in the first group 0.27(0–1) and 0.27 (0–2) (U = 60.5, *p* > 0.05). One-year control examination has shown the absence of exophthalmos and differences between groups (0 and 0), but positive dynamics inside groups (*p* < 0.05).

Before operation, the appearance of exophthalmos in third and fourth group was approximately equal (1.54; 0–3 and 1.36; 0–3) (U = 21, *p* < 0.01). In 30 days after operation, the means of the exophthalmos test were different, but not significantly, in both groups (0.09(0–1) and 0) (U = 38, *p* > 0.05). One-year control examination has shown the absence of exophthalmos and differences between groups (0 and 0), but positive dynamics inside groups (*p* < 0.05).

Before operation, the appearance of enophthalmos in first and second group was approximately equal (0.27; 0–2 and 0.36; 0–2) (U = 55.5, *p* > 0.05). In 30 days after operation, the means of enophthalmos test were less in the first group (0 and 0.54; 0–2) (U = 33, *p* < 0.05). One-year control examination has shown the absence of enophthalmos in the first group, and its increase in the second group (0 and 0.9; 0–3) (U = 22, *p* < 0.01), with negative dynamics (*p* > 0.05).

Before operation, the appearance of enophthalmos in third and fourth group was approximately equal (0.09; 0–1 and 0.18; 0–1) (U = 55, *p* > 0.05). In 30 days after operation, the means of enophthalmos test were less in the third group (0 and 0.36:0–1) (U = 38.5, *p* > 0.05). One-year control examination has shown the absence of enophthalmos in the first group, and its increase in the second (0 and 1.18; 0–3) (U = 11, *p* < 0.11), with negative dynamics inside the last (ps. 0.05).

Before operation, the presence of diplopia in first and second group was approximately equal (0.55; 0–1 and 0.45; 0–1) (U = 55.5, *p* > 0.05). In 30 days after operation, the means of diplopia test were less in the first group (0.18; 0–1 and 0.27; 0–1) (U = 55, *p* > 0.05). A one-year control examination has shown the absence of diplopia in the first group and its increase in the second group (0 and 0.45; 0–1) (U = 33, *p* < 0.05) with negative dynamics (*p* > 0.05).

Before operation, the presence of diplopia in third and fourth group was approximately equal (0.55; 0–1 and 0.45; 0–1) (U = 55.5, *p* > 0.05). In 30 days after operation, the means of diplopia test were less in the third group (0 and 0.45; 0–1) (U = 33, *p* < 0.05). One-year control examination has shown the absence of diplopia in the third group, and its increase in the fourth group (0 and 0.64; 0–1) (U = 22, *p* < 0.01), with negative dynamics (*p* > 0.05).

Comparing different symptoms for search of correlation, we found a strong positive connection between diplopia and enophthalmos in the fourth group (r = 0.51, *p* = 0.0025), medium correlation between diplopia and enophthalmos in the first group (r = 0.41, *p* = 0. 014) and in the third group (r = 0.38, *p* = 0.032) and a weak correlation between these criteria in the second group (r = 0.25, *p* = 0.16). The density of this connection was different according to groups: in the first and third group, it was almost average (rs = 0.45, *p* = 0.0088 and rs = 0.38, *p* = 0.032); in the fourth group, it was high (rs = 0.54, *p* = 0.0013); and in the second group, it was low (rs = 0.2, *p* = 0.27).

Results are demonstrated in Table S1A (Supplementary Materials).

## 4. Discussion

All patients in this study had preoperative and postoperative clinical examination, as well as radiographs and CT scans data were analysed. As a result of zygomatic bone displacement and rotation, it was found that majority of patients (n = 56, 46.67%) had a linear fracture occur in the fronto-zygomatic suture area, in combination with linear or comminuted fractures of the infraorbital rim and zygomatico-alveolar buttress. According to Manson et al.’s classification [21] middle-energy and high-energy zygomatic complex fractures were more prevalent.

Orbital fractures with various degrees of comminution and defect size are almost always seen in patients with zygomatic complex fractures [22]. According to our data, fractures of the zygomatic complex in 34 (28.33%) cases required orbital floor reconstruction, due to significant injury. Wilde et al. in their study showed that only two of 19 (10%) zygomaticomaxillary complex fractures required orbital floor reconstruction, according to intraoperative computed tomography findings after zygomatic bone reduction [23]. Flynn and colleagues demonstrated that, of 1396 patients who underwent operative reduction, 23% of these patients underwent concomitant orbital floor repair [24].

Different degrees of the eye globe contusions were detected in 38 (86.33%) cases. Diplopia or oculomotor function disorders were diagnosed in 20 (45%) patients. B. Evans and G. Evans (2008) observed diplopia in 60% of the examined patients [25]. At the same time, the authors note that these symptoms persisted for three months after surgery in 5% of cases, but signs of diplopia were not determined after six months. In the study performed by Salma et al., post-traumatic diplopia was diagnosed in 59% of patients. Only 13% of cases had pre-operative diplopia. Post-surgical diplopia was reported in 37% of cases. Authors conclude that traumatic diplopia in unilateral middle- and high-energy zygomaticomaxillary complex fractures is a common symptom [26].

Sensory disorders in the infraorbital nerve system are common after zygomatic complex and orbital floor fractures. In most cases, the recovery of pain and tactile sensitivity in the II branch of the trigeminal nerve occurred within 3–6 months. Persistent disorders of the infraorbital nerve were noted in two (5%) cases. In the study performed by Homer at al. A total of 20 of 42 patients (47.6%) with zygomatic complex and isolated orbital fractures had some infraorbital hypesthesia. There were 31.8% with isolated orbital floor fractures versus 68.4% zygomaticomaxillary complex fractures. The authors concluded that patients with zygomaticomaxillary complex fractures showed no difference in sensory prognosis between those repaired and observed [27].

In patients with zygomatic complex fractures with a displacement of bony fragments, osteosynthesis was performed at three, two and one point in 57 (47.5%), 56 (46.67%) and seven (5.83%) cases, respectively. The choice of a fixator was carried out in accordance to its biomechanics, fracture localisation and pattern. Gadkari et al. in their systematic review concluded that a 3-point fixation is superior to a 2-point fixation in reducing malar asymmetry in zygomaticomaxillary complex fractures [28]. It should be emphasised that zygomatico-alveolar buttress is the optimal point for fracture fixation. We adhere to this point of view, and find that osteosynthesis in this area, if possible, should be performed in all patients.

Endoprosthesis of the lower orbital wall of titanium nickelide mesh was performed in 11 patients with fractures of the zygomatico-orbital complex (group 1) and 11 patients with blow-out fractures (group 2). The use of this material restores the anatomy of the lower wall of the orbit with a good result. The mesh structure allows tissues to penetrate through the implant with good fixation, preventing displacement. The use of super-elastic titanium nickelide implants increases the efficiency of surgical treatment of patients with orbital floor fractures. Shtin et colleagues emanating from their clinical observations claim that the knitted TiNi-based mesh indicates a high level of biocompatibility. It is simply modified intraoperatively, to attach any desired shape/size for implantation, and can also be screw-fixed, providing a good supporting ability. The authors concluded that this method of reconstruction may be an attractive alternative to Ti-based meshes and plates in patients with enophthalmos, hypoglobus or diplopia, with good functional and cosmetic outcomes [17].

According to the literature, titanium mesh is an optimal material to reconstruct defects in the treatment of orbital wall fractures. On the other hand, its stiffness can be a disadvantage in cases of recurrent facial trauma, which may cause mesh distortion and injury to orbital structures [29].

It was found that the use of mini clamps made of titanium nickelide alloy is to minimise surgical trauma by reducing the length of the lateral eyebrow skin incision and decreasing the number of drilled holes in the bone. Other advantages reported by Strackee et al. were that staple fixation has a minimal effect on the periosteal blood supply, and little dissection is necessary to place drill holes. A characteristic of the titanium nickelide alloy is the shape-memory effect: its shape is temperature dependent. The fixation material has less bulk compared to plates, thus reducing the need for its removal in case of dental implants [30]. In cases of zygomatic complex fractures, which are not accompanied with a bony defect at the area of the infraorbital rim, it is recommended to perform osteosynthesis with ellipsoid titanium nickelide mini clamp via a vestibular approach. In addition, this technique allows to reduce the number of foreign bodies and their mass in the organism. The miniature size of these fixators, as well as their ease of installation, makes this method promising for use in maxillofacial surgery for osteosynthesis in zygomatic complex fractures.

Great interest in the creation of metal implants came about in the period of the late nineteenth to early twentieth century. High-grade steel implants have been used in medical clinics. Fixation methods using nickel-plated plates and steel screws have been developed for the treatment of bone fractures. However, the plates were often destroyed, and their effectiveness decreased. High-strength vanadium steel was later used for the manufacture of trauma plates and clamps. Analysis of the properties of metallic materials and alloys shows that alloys based on nickel-titanium ones are the most compatible materials with body tissues, which is due to the delay of biological systems [4].

The hysteretic behaviour of the alloys based on titanium nickelide of the TH-10 type at a temperature of +36 °C corresponds to the hysteretic delayed reaction of tissues. Correction of the hysteresis width and the value of the reversible deformation εcr can be carried out by changing the composition of the TN-10 alloy and thermomechanical processing. Therefore, for the corresponding fabrics with a certain hysteresis width and strain, it is necessary to select an alloy based on TH-10 with the given parameters of the hysteresis width and the reversible strain value. The achievement of such predetermined conditions poses the problem of biomechanical compatibility of implants and tissues to a qualitatively new level. Coordination of new directions in the creation of medical materials, tools and implants of a new generation is carried out at the Research Institute of Medical Materials and Implants with shape memory (Tomsk), together with various medical institutions in Russia [31].

## 5. Conclusions

The use of individual titanium nickelide implants increased the effectiveness of surgical treatment of patients with orbital floor fractures. High bio inertness, possibility of intraoperative implant modelling, elasticity, good restoration of the anatomical contour of orbit and the absence of fixation reduce the duration of surgery and the rehabilitation period. Such treatment tactics in the early period after injury prevents post-traumatic deformities and visual impairment.

## 6. Patents

‘Method for restoring the lower wall of the orbit and lower eye region after injury’ RU-2-486-872-C1-10.07.2013.

## Figures and Tables

**Table 1:** Characteristics of four groups with trauma on admission.

Group	Age, Years Mean	Cause, Points Mean	Patient Admission, Days Mean
	(Median, Min–Max)	(Median, Min–Max)	(Median, Min–Max)
	Standard Error	Standard Error	Standard Error
Group 1	30.2	0.8	2.1
	(30, 18–45)	(1, 0–1)	(2, 1–5)
	2.8	0.12	0.37
Group 2	33.3	0.9	2.2
	(34, 20–47)	(1, 0–1)	(2, 1–4)
	2.6	0.09	0.3
Group 3	31.3	0.55	1.8
	(31, 19–46)	(1, 0–1)	(2, 1–3)
	2.5	0.16	0.23
Group 4	30.8	0.64	1.9
	(31, 18–47)	(1, 0–1)	(2, 1–5)
	3	0.15	0.37
*p*	0.82	0.2	0.8

## Supplementary Materials

The following are available online at https://www.mdpi.com/2079-4983/11/3/52/s1, Table S1A: Main characteristics of the research in dynamics.
