# Supplementary material for: Titanium Nickelide in Midface Fractures Treatment
Source: J Funct Biomater. 2020 Jul 27;11(3):52. doi: 10.3390/jfb11030052 (PMC7564408; doi:10.3390/jfb11030052)
Supplement: Supplementary file 1 [file jfb-11-00052-s001.pdf]

Supplementary Materials: Titanium Nickelide in Midface Fractures Treatment

Liudmila Shamanaeva [1,*], Ekaterina Diachkova [2], Pavel Petruk [1], Kirill Polyakov [1], Igor Cherkesov [1] and Sergei Ivanov [1,3]

Table S1A. Main characteristics of the research in dynamics.

**Period**	**Paresthesia _1gr**<br>**Mean±SD (Median, Min-Max)**<br>**Standard error**	**Paresthesia _2gr**<br>**Mean±SD (Median, Min-Max)**<br>**Standard error**	**Exophtal mos_1gr**<br>**Mean±SD (Median, Min-Max)**<br>**Standard error**	**Exophtalm os_2gr**<br>**Mean±SD (Median, Min-Max)**<br>**Standard error**	**Exophtalm os_3gr**<br>**Mean±SD (Median, Min-Max)**<br>**Standard error**	**Exophtalm os_4gr**<br>**Mean±SD (Median, Min-Max)**<br>**Standard error**	**Enophtal mos_1gr**<br>**Mean±SD (Median, Min-Max)**<br>**Standard error**	**Enophtal mos_2gr**<br>**Mean±SD (Median, Min-Max)**<br>**Standard error**	**Enophtal mos_3gr**<br>**Mean±SD Median, (Min-Max)**<br>**Standard error**	**Enophtal mos_4gr**<br>**Mean±SD (Median, Min-Max)**<br>**Standard error**	**Diplopia_ 1gr**<br>**Mean±SD (Median, Min-Max)**<br>**Standard error**	**Diplopia_ 2gr**<br>**Mean±SD (Median, Min-Max)**<br>**Standard error**	**Diplopia_ 3gr**<br>**Mean±SD (Median, Min-Max)**<br>**Standard error**	**Diplopia_ 4gr**<br>**Mean±SD (Median, Min-Max)**<br>**Standard error**
before	1.09±0.54<br>(1,0-2)<br>0.16	1 ±0.65<br>(1,0-2)<br>0.23	1.36±1.03<br>(1,0-3)<br>0.31	1.09±0.97<br>(1,0-3)<br>0.28	1.54±0.95<br>(1,0-3)<br>0.28	1.36±0.93<br>(1,0-3)<br>0.28	0.27±0.8<br>(0,0-2)<br>0.19	0.36±0.81<br>(0,0-2)<br>0.2	0.09±0.88<br>(0,1.0-1)<br>0.09	0.18±0.88<br>(0,0-1)<br>0.12	0.55±0.52<br>(1,0-1)<br>0.16	0.45±0.5<br>(0,0-1)<br>0.16	0.55±0.52<br>(1,0-1)<br>0.16	0.45±0.51<br>(0,0-1)<br>0.16
Post-operative 30d	0.55±0.65<br>(1,0-1)<br>0.16	0.55±0.67<br>(0,0-2)<br>0.2	0.27±0.95<br>(0,0-1)<br>0.14	0.27±0.89<br>(0,0-1)<br>0.14	0.09±0.9<br>(0,0-1)<br>0.09	0	0	0.54±0.81<br>(0,0-2)<br>0.2	0	0.36±0.69<br>(0,0-1)<br>0.15	0.18±0.49<br>(0,0-1)<br>0.12	0.27±0.49<br>(0,0-1)<br>0.14	0	0.45±0.51<br>(0,0-1)<br>0.16
Post-operative 365d	0.09±0.67<br>(0,0-1)<br>0.09	0.09±0.66<br>(0,0-1)<br>0.09	0	0	0	0	0	0.9±0.88<br>(1,0-3)<br>0.28	0	1.18±0.8<br>(1,0-3)<br>0.26	0	0.45±0.49<br>(0,0-1)<br>0.16	0	0.64±0.51<br>(1,0-1)<br>0.15
p	>0.05		>0.05		>0.05		**<0.01**		**<0.01**		>0.05		**<0.05**	
